# Functional analysis of stress protein data in a flor yeast subjected to a biofilm forming condition

**DOI:** 10.1016/j.dib.2016.03.072

**Published:** 2016-03-28

**Authors:** Jaime Moreno-García, Juan Carlos Mauricio, Juan Moreno, Teresa García-Martínez

**Affiliations:** a Department of Microbiology, Severo Ochoa (C6) Building, Agrifood Campus of International Excellence ceiA3, University of Cordoba, Ctra. N-IV-A, Km 396, 14014 Cordoba, Spain; b Department of Agricultural Chemistry, Marie Curie (C3) Building, Agrifood Campus of International Excellence ceiA3, University of Cordoba, Ctra. N-IV-A, Km 396, 14014 Cordoba, Spain

## Abstract

In this data article, an OFFGEL fractionator coupled to LTQ Orbitrap XL MS equipment and a SGD filtering were used to detect in a biofilm-forming flor yeast strain, the maximum possible number of stress proteins under the first stage of a biofilm formation conditions (BFC) and under an initial stage of fermentation used as reference, so-called non-biofilm formation condition (NBFC). Protein functional analysis – based on cellular components and biological process GO terms – was performed for these proteins through the SGD Gene Ontology Slim Mapper tool. A detailed analysis and interpretation of the data can be found in “Stress responsive proteins of a flor yeast strain during the early stages of biofilm formation” [1].

## 1 **Specifications Table**

**Table:** 

Subject area	*Biology, Microbiology and Biochemistry*
More specific subject area	*Proteomics, Bioinformatics*
Type of data	*Tables*
How data was acquired	*Protein identification: 3100 OFFGEL Fractionator (Agilent Technologies, Palo Alto, CA) and LTQ Orbitrap XL mass spectrometer (Thermo Fisher Scientific, San Jose, CA, USA) equipped with a nano LC Ultimate 3000 system (Dionex, Germany); Protein quantification: emPAI; Bioinformatic analyses: SGD*
Data format	*Filtered and analyzed*
Experimental factors	*Saccharomyces cerevisiae G1 flor yeast strain; biofilm formation condition and non-biofilm formation condition as control; bioinformatic analyses of the stress response-related identified proteins*
Experimental features	*Saccharomyces cerevisiae G1 flor yeast was exposed to a biofilm formation condition with ethanol and glycerol as main carbon sources and non-biofilm formation condition with glucose. After protein fragmentation using OFFGEL and the identification of the stress response-related proteins, bioinformatic analyses were performed to investigate the protein sub-cellular localization and biological functions*
Data source location	*Agrifood Campus of International Excellence ceiA3, University of Cordoba, Spain*
Data accessibility	*Data are within this article*

## 2 **Value of the data**

• Through the SGD Gene Ontology Slim Mapper tool, flor yeast stress proteins were sorted in cellular components and biological GO Terms. Comparison among stress and non-stress conditions of protein frequency sorted in each term, allows to highlight relevant GO Terms.
• The bioinformatics tools applied in this study allows to interpret biological information to be used for comparative proteomics studies.
• The association of proteomic data with protein activity assays and genetics may lead to the genetic improvement of flor yeast strains.

## 3 Data

Here, we show sub-cellular localizations (Table 1 in supplementary data) and biological processes (Table 2 in supplementary data) GO Terms in which the flor yeast stress related-proteins detected in stressed biofilm formation condition (BFC) and non-biofilm formation condition (NBFC) were sorted. Each type of biofilm formation stresses (lack of fermentable carbon source, ethanol, acetaldehyde and oxidative) were considered separately. Comparison with the *Saccharomyces cerevisiae* proteome frequency, *p*-value and the “GO Term frequency BFC/GO Term frequency NBFC” ratio highlighted most relevant cellular components and biological processes in each condition.

## 4 Experimental design, materials and methods

The effects of two different biofilm formation conditions (BFC and NBFC) on *S. cerevisiae* G1 flor yeast stress response related-protein expression patterns have been analyzed by using an offgel-based approach. Culture conditions were performed as described in the Process Biochemistry journal paper [1]. Briefly, after growing until the yeast viability reached 90% at the exponential phase, under the two different conditions: BFC with ethanol and glycerol and NBFC with glucose as the main carbon sources; yeasts were collected and proteins extracted. In both conditions, for triplicates, three aliquots for proteomic analysis were carried out. OFFGEL fractionation, LTQ Orbitrap XL mass spectrometer identification, emPAI quantification [2] and SGD filtration were used to obtaining the stress response proteins in each condition. Bioinformatic tool Gene Ontology Slim Mapper from SGD (http://www.yeastgenome.org/), were applied in order to clarify the sub-cellular localization and biological processes of the identified proteins.

## Footnotes

Appendix A Supplementary data associated with this article can be found in the online version at 10.1016/j.dib.2016.03.072.
