# Supplementary material for: Functional analysis of stress protein data in a flor yeast subjected to a biofilm forming condition
Source: Data Brief. 2016 Mar 28;7:1021–3. doi: 10.1016/j.dib.2016.03.072 (PMC4826585; doi:10.1016/j.dib.2016.03.072)
Supplement: Supplementary file 1 — Supplementary material [file mmc1.docx]

The authors declare no conflict of interest form
